# Inhibition of host cell division by T5 protein 008 (Hdi)

**DOI:** 10.1128/spectrum.01697-23

**Published:** 2023-10-27

**Authors:** Tridib Mahata, Shahar Molshanski-Mor, Moran G. Goren, Miriam Kohen-Manor, Ido Yosef, Oren Avram, Dor Salomon, Udi Qimron

**Affiliations:** 1 Department of Clinical Microbiology and Immunology, School of Medicine, Tel Aviv University, Tel Aviv, Israel; 2 The Shmunis School of Biomedicine and Cancer Research, George S. Wise Faculty of Life Sciences, Tel Aviv University, Tel Aviv, Israel; Wuhan University, Wuhan, China

**Keywords:** host takeover, bacterial division, bacteriophage biology

## Abstract

Bacteriophages (phages) have evolved effective methods of exploiting host-cell machinery by redirecting molecular pathways. In this study, we describe an inhibitor of bacterial growth, T5.008, encoded by the T5 phage. High-throughput sequencing of genomic DNA from bacterial mutants resistant to T5.008 identified mutations in the *Escherichia coli ftsZ* gene, suggesting that T5.008 targets the cell division protein FtsZ. We validated this finding by replacing a residue in FtsZ with one that confers protection from T5.008 inhibition. We used time-lapse microscopy to show that T5.008 interferes with FtsZ ring formation and stability, thereby inhibiting division. We also demonstrated that phages expressing T5.008 can prevent host division before lysis much more efficiently than those lacking T5.008. We also showed that T5.008 provides a competitive advantage to the T5 phage. This strategy of inhibiting division during phage infection likely enables T5 phage to utilize host-cell resources efficiently.

**IMPORTANCE:**

We have identified a novel phage-encoded inhibitor of the major cytoskeletal protein in bacterial division, FtsZ. The inhibition is shown to confer T5 bacteriophage with a growth advantage in dividing hosts. Our studies demonstrate a strategy in bacteriophages to maximize their progeny number by inhibiting escape of one of the daughter cells of an infected bacterium. They further emphasize that FtsZ is a natural target for bacterial growth inhibition.

## INTRODUCTION

Phages are viruses that infect bacteria and have coevolved with them for extended periods. As a result, phages have developed specific mechanisms for inhibiting or altering critical metabolic processes in their host bacteria. Increasing our understanding of the pathways targeted by phages and the gene products used to disrupt these pathways could create new tools for manipulating bacteria. In the last few years, we have identified several phage products that regulate bacterial metabolism: the 0.4 protein of phage T7 inhibits the essential cell division protein FtsZ (1); the 0.6 protein of phage T7 inhibits the essential cytoskeletal protein MreB (2); the 015 protein of phage T5 cleaves DNA at abasic sites along with the DNA repair protein Ung (3). Many more phage products are likely to interact with as-yet unidentified bacterial targets. We believe that searching for them will expand our knowledge, increase the repertoire of bacterial targets, and help in the fight against bacterial infections.

*Escherichia coli* serves as an invaluable model for investigating host-virus interactions and exploring phage-based antibacterial approaches. The laboratory strain of *E. coli* K-12 shares critical genes with pathogenic strains like *E. coli* O157:H7 and O104:H4, implying that growth inhibitors targeting K-12 could be effective against these pathogens. Comprehensive research has been conducted on *E. coli*, leading to the identification of putative functions or potential physiological roles for over half of its 4,453 genes.

Among the phages studied, T5 is a lytic phage capable of causing host cell lysis upon a successful growth cycle. Numerous gene products of T5 have been extensively characterized, including its structural components and those involved in DNA replication. However, the precise mechanism by which T5 manipulates host functions remains unclear.

To address this knowledge gap, we have postulated that some of the uncharacterized gene products of T5, along with their interactions with host proteins, may be responsible for inhibiting *E. coli* growth through the targeted modulation of specific host proteins or pathways.

Previously, we developed a technology utilizing whole-genome DNA sequencing (2) to identify antibacterial targets of phage proteins. The underlying principle of this method lies in the detection of mutations that provide resistance to growth inhibitors, which can indicate the target genes. By expressing a growth inhibitor and employing whole-genome sequencing, we can pinpoint the specific mutations responsible for conferring resistance.

Advancements in DNA-sequencing technology, coupled with increased accessibility and affordability, have rendered this approach cost-effective for identifying bacterial targets. In this study, we applied this technology to investigate bacterial targets of phage proteins, leading to the discovery of an interaction between an inhibitory T5 phage protein and the *E. coli* cell division protein, FtsZ.

The bacterial cell division process is an intricate and precisely regulated event crucial for the propagation and proliferation of bacterial populations. Central to this process is the protein FtsZ, a highly conserved and fundamental component in bacterial cytokinesis (4). FtsZ serves as a key organizer for the formation of the Z-ring, a dynamic structure that assembles at the division site and orchestrates the recruitment of various cell division proteins (5). Through its GTPase activity, FtsZ exhibits polymerization and depolymerization dynamics, enabling it to assemble into filaments, known as the protofilaments, which then further interact to form the Z-ring (6). The spatial and temporal regulation of FtsZ, along with its interactions with other division proteins, is essential for coordinating the septal constriction and subsequent separation of daughter cells (7). Due to its pivotal role in bacterial cell division, FtsZ has emerged as a prime target for antimicrobial drug development, underscoring the significance of further research to unravel its intricate molecular mechanisms and regulatory functions.

In this work, we demonstrate that a gene of T5 phage destabilizes the FtsZ rings, leading to division inhibition of the bacteria. We show that this activity provides a competitive advantage to the phage and speculate that it does so by preventing bacterial daughter cells from escaping infection by the phage.

## RESULTS

### Gene 008 of phage T5 inhibits growth of different bacterial strains

**Fig 1:**
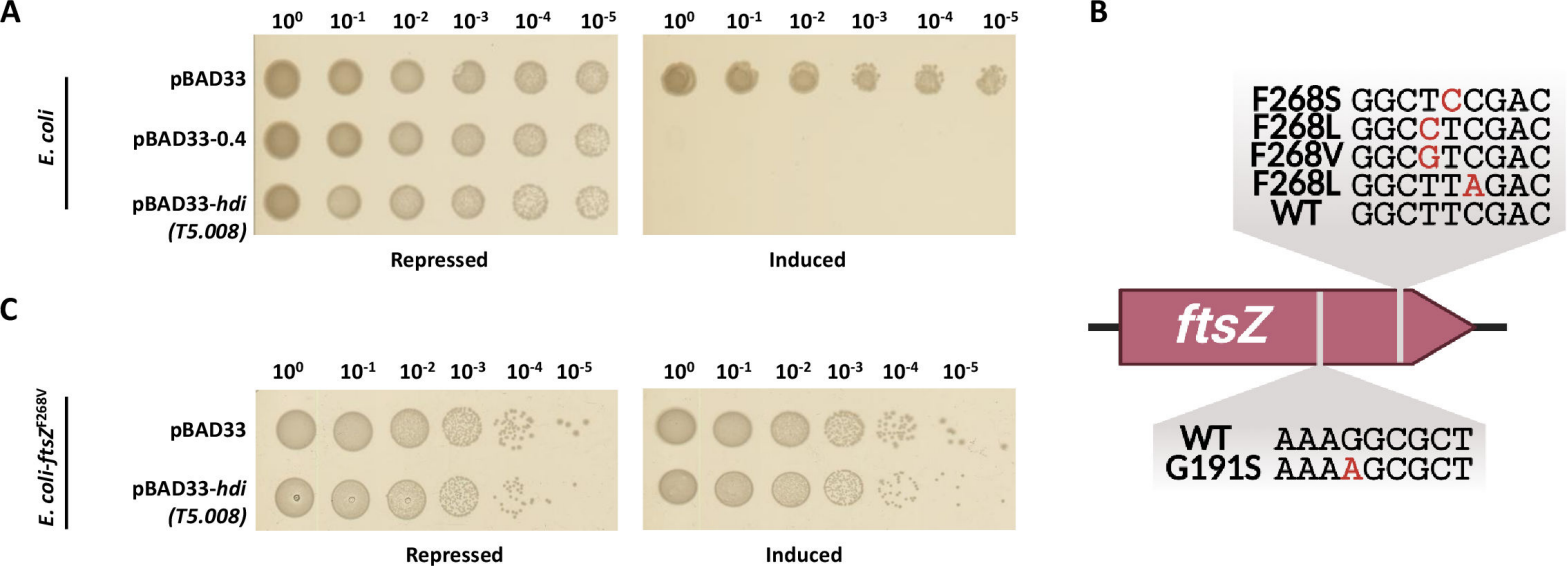
T5.008 inhibition of *E. coli* growth. (**A**) *E. coli* NEB5α transformed with a plasmid encoding the indicated phage gene or with the control pBAD33 vector was inoculated on LB agar plates supplemented with 0.2% D-glucose (Repressed) or 0.2% L-arabinose (Induced). (**B**) Schematic representation of the mutations identified as resistant to T5.008 inhibition. (**C**) NEB5α encoding FtsZ^F268V^ transformed with a plasmid encoding the indicated phage gene or with the control pBAD33 vector was inoculated on LB agar plates supplemented with 0.2% D-glucose (Repressed) or 0.2% L-arabinose (Induced). Results of one representative experiment out of three are shown.

We previously reported that T5 gene 008 (T5.008) inhibits the growth of *E. coli* (3). We cloned it with its natural ribosome-binding site into plasmid pBAD33 under the tightly regulated pBAD promoter (8). *E. coli* NEB5α cultures harboring the pBAD33 vector as a negative control, Gp0.4 from T7 phage as a positive control killing the bacteria by inhibiting the division protein FtsZ (1), and T5.008 were serially diluted and inoculated on LB agar plates with or without the inducer L-arabinose. The negative control grew on both plates, whereas T5.008 and the positive control grew only on the plates without an inducer, indicating that, similar to the positive control, the T5.008 product inhibits *E. coli* growth (Fig. 1A). We further showed that T5.008 inhibited three other bacteria species: *Salmonella enterica*, *Shigella sonnei*, and *Enterobacter cloacae* (Fig. S1). These results indicated that although derived from a coliphage, the T5.008 product also inhibits other bacterial species.

### Isolation of *E. coli* mutants resistant to T5.008

To identify the target of T5.008, we plated ~10^9^ bacteria harboring the T5.008-encoding plasmid under inductive conditions and isolated resistant mutants. Such mutants may arise from genomic mutations in the target of T5.008 or from plasmid loss or mutations in T5.008 that render it ineffective. To exclude the two latter possibilities, we extracted the plasmids from resistant colonies and validated their toxicity by transforming them into naïve bacterial cells. We identified seven clones containing plasmids that retained toxicity, indicating that the clones harbor genomic mutations conferring resistance to T5.008. These clones were used in subsequent analyses.

### Mutations in *ftsZ* eliminate T5.008-mediated toxicity

The genomic DNA of all seven mutants resistant to the expression of T5.008 was sequenced in an Illumina-based high-throughput sequencer. The results yielded ~30× coverage of each genome, sufficient to detect single-nucleotide polymorphisms with a high confidence score (1, 2). The deep DNA-sequencing analysis revealed that the *ftsZ* gene, encoding the FtsZ cell division protein, was specifically mutated in all of the T5.008-resistant clones, resulting in substitution of two amino acids, G191 and F268 (Fig. 1B; Table S1). The mutations were then confirmed using Sanger sequencing. To further validate these results, we introduced one of the identified mutations, FtsZ^F268V^, into naïve *E. coli*, and tested its resistance to T5.008. *E. coli* cultures encoding FtsZ^F268V^ were transformed with either the inducible T5.008 plasmid or with the pBAD33 control vector. Serially diluted cultures were then inoculated on LB agar plates with or without the inducer L-arabinose. We observed that *E. coli* encoding the FtsZ^F268V^ variant was resistant to T5.008 as it showed similar growth in the presence of T5.008 or the pBAD33 vector (Fig. 1C). These results show that T5.008 inhibits FtsZ, but not the FtsZ^F268V^ variant. We consequently renamed the T5.008 protein: Host division inhibitor (Hdi).

### Not all substitutions of F268 resist toxicity

**Fig 2:**
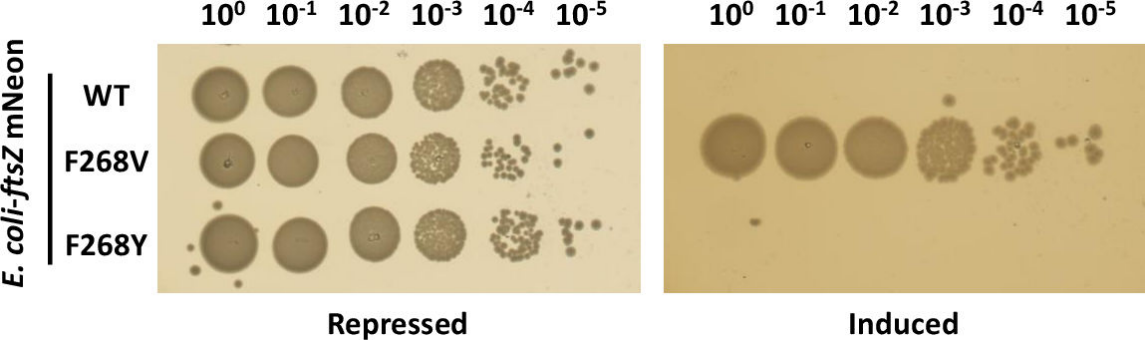
Aromatic residue at position 268 in FtsZ maintains sensitivity to Hdi. (**A**) *E. coli* K-12-ftsZ mNeon encoding the indicated FtsZ and transformed with a plasmid encoding Hdi was inoculated on LB agar plates supplemented with 0.2% D-glucose (Repressed) or 0.2% L-arabinose (Induced). Results of one representative experiment out of three are shown.

We identified three different substitutions at position 268 of FtsZ—valine, serine, and leucine—that resisted inhibition by Hdi. To examine whether the phenylalanine residue is crucial for inhibition, we mutated residue 268 to another aromatic residue, tyrosine, and tested the inhibition by inducing Hdi in these cells. Hdi did not inhibit the growth of bacteria encoding F268V, but it did inhibit the growth of FtsZ with the F268Y substitution, similar to the positive control wild-type FtsZ (Fig. 2). These results indicated that phenylalanine is not the only residue at this position that allows Hdi-mediated inhibition of FtsZ.

### Hdi destabilizes the FtsZ rings during bacterial growth

**Fig 3:**
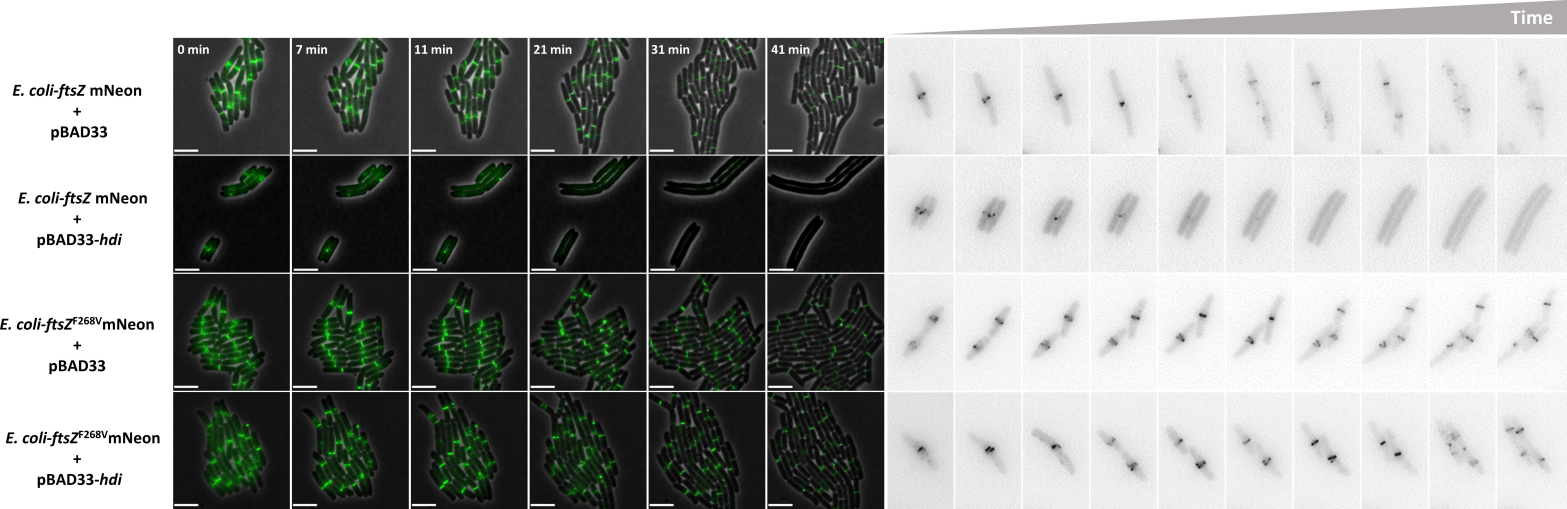
Time-lapse microscopy of bacteria encoding fluorescent FtsZ. *E. coli* K-12-ftsZ mNeon encoding the indicated FtsZ and harboring the indicated plasmid was induced with 0.01% L-arabinose at time = 0 min and spotted on agarose pads. Time-lapse imaging was carried out as described in the Experimental Procedures for the indicated time (left). The Z ring of individual cells is shown as black against a white background (right). Scale bar = 5 µm.

To understand the effect of Hdi on FtsZ and cell division, we used high-resolution microscopy to produce real-time images of the FtsZ ring during bacterial growth and division. We used bacteria encoding a functional FtsZ tagged with mNeon (9) and followed their growth. The bacteria transformed with a control vector manifested distinct FtsZ rings and normal division. In contrast, bacteria transformed with a plasmid encoding Hdi manifested disappearance of the detectible rings within ~15 min, and we observed bacterial elongation rather than cell division (Fig. 3; Movies S1 and S2). In bacteria encoding FtsZ^F268V^, resistant to Hdi inhibition, we observed distinct FtsZ rings and normal division in cells transformed with either a control vector or plasmid encoding Hdi (Fig. 3; Movies S3 and S4). The average length of individual bacteria encoding the wild-type FtsZ in the presence of Hdi was significantly higher than the control whereas those encoding FtsZ^F268V^ showed similar length (Fig. S2). These results demonstrated that the Hdi protein destabilizes the wild-type FtsZ but not FtsZ^F268V^ rings, thus inhibiting cell division in sensitive bacteria.

### Hdi destabilizes FtsZ rings also during phage infection

**Fig 4:**
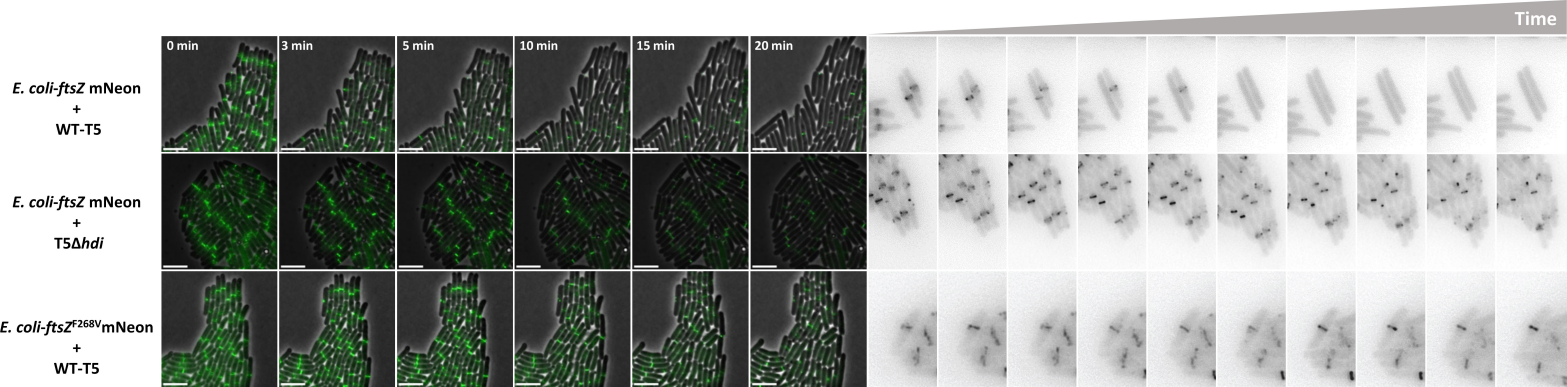
Time-lapse microscopy of T5-infected bacteria encoding fluorescent FtsZ. *E. coli* K-12-ftsZ mNeon encoding the indicated FtsZ was infected with the indicated phage at time = 0 min and spotted on agarose pads. Time-lapse imaging was carried out as described in the Experimental Procedures for the indicated time (left). The Z ring of individual cells is shown as black against a white background (right). Scale bar = 5 µm.

We next infected bacteria encoding GFP-tagged FtsZ with T5 phages and monitored the FtsZ rings. Within 10 min of infection with the wild-type T5 encoding *hdi*, distinct FtsZ rings were eliminated from ~75% of the bacterial population (Fig. 4; Movie S5). In contrast, when a T5 lacking *hdi* (T5Δ*hdi*) was used to infect the bacteria, only ~25% of the bacterial population lost the FtsZ rings within 10 min of infection (Fig. 4; Movie S6). Moreover, when wild-type T5 was used to infect bacteria with GFP-tagged FtsZ^F268V^, only ~6% of the bacterial population lost the FtsZ rings within 10 min of infection (Fig. 4; Movie S7). The residual FtsZ ring loss in the absence of Hdi activity might have been due to the effect of nonspecific phage protease activity (10, 11). These results clearly showed that the physiological role of Hdi during T5 phage growth is to destabilize the FtsZ rings.

### Hdi confers a competitive advantage to phage T5 by inhibiting cell division

**Fig 5:**
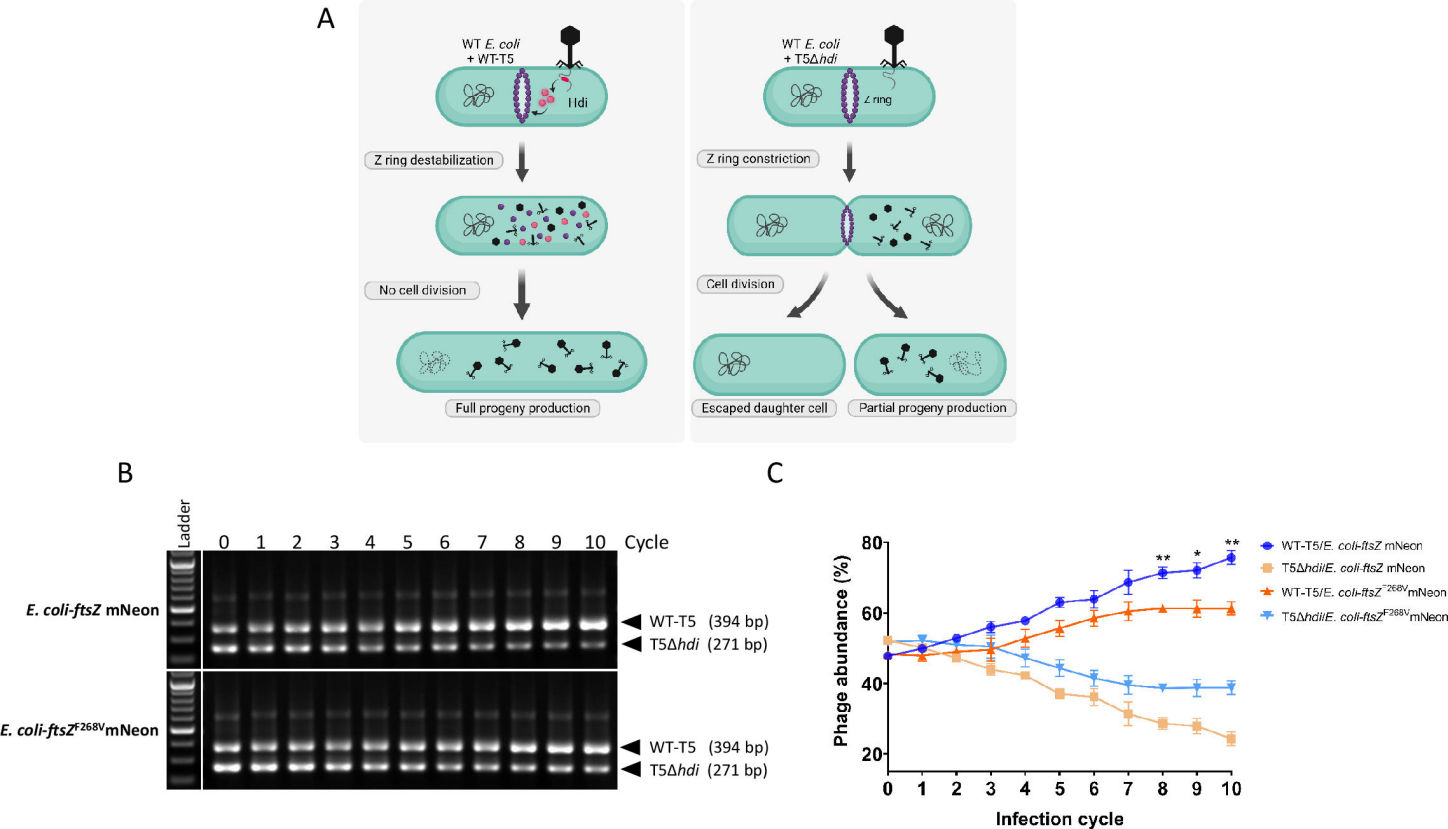
PCR-quantified competition between phages. (**A**) A model illustrating the competitive advantage gained from preventing the escape of a daughter cell. (**B**) PCR amplifying the region flanking the *hdi* gene was carried out on wild-type T5 phage and T5Δ*hdi* mixed at similar ratios to a multiplicity of infection (MOI) of ~0.1. The upper bands in the gel images are products obtained from amplifying the DNA of wild-type T5, and the lower bands are from T5Δ*hdi*. (**C**) Graphs showing the abundance of the indicated phages grown on the indicated hosts. The PCR and abundance calculations were carried out as described in the Experimental Procedures. Graphs show average ± SD of three independent experiments. * *P* < 0.05 and **P < 0.001.

Finally, we hypothesized that the physiological role of Hdi is to confer a competitive advantage to the phage infecting a dividing cell. If a cell divides early in infection, while containing only a single phage genome, one daughter cell will escape and phage propagation will be confined to only half of the cell resources. Hdi inhibition of daughter cell escape allows the phage’s progeny to have all of the cell resources at their disposal (Fig. 5A). To test this hypothesis, we used T5Δ*hdi* and compared its competitive ability against the wild-type T5 in hosts encoding a wild-type copy of FtsZ. To determine the relative abundance of T5Δ*hdi* compared with wild-type T5 in a phage mixture, we used PCR to amplify the region flanking the *hdi* gene. This PCR discriminates between the two phages because amplification of the wild-type T5 phage results in a longer product than amplification of the deletion mutant. This assay is quantitative, enabling detection of different ratios of phage mixtures (Fig. S3). We used a mixture containing an equal ratio of wild-type T5 to T5Δ*hdi* to infect *E. coli* hosts. Phage lysates were collected after each of 10 consecutive infection cycles, and PCR was carried out on these samples to measure the ratio of each phage (Fig. 5B). Quantification of the bands’ intensities showed that the wild-type T5 has a significant growth advantage compared with T5Δ*hdi*, indicating that Hdi significantly increases the competitiveness of the wild-type T5 phage (Fig. 5C). We further used *E. coli* encoding FtsZ^F268V^—the variant identified as resistant to Hdi inhibition—as a host. We postulated that in this host, the competitive advantage of wild-type T5 would be reduced, because FtsZ^F268V^ is less inhibited. Indeed, on FtsZ^F268V^-encoding hosts, wild-type T5 had a lower competitive advantage compared with its advantage on wild-type FtsZ-encoding hosts (Fig. 5A and B). These results were further validated by sampling individual plaques from the initial and final cycles of the competition on the two hosts and genotyping them by PCR flanking the *hdi* deletion to determine the fraction of the wild-type and the deletion mutant phages at each cycle. Samples of wild-type T5 in the initial cycle accounted for 13/30 plaques; after 10 competition cycles on the K-12-ftsZ mNeon, the sample count was 27/32 plaques; on the K12-ftsZ^F268V^ mNeon, the sample count was 21/32 after 10 competition cycles (Fig. S4). Although not statistically significant, the wild-type T5 still retained some competitive advantage, perhaps because FtsZ^F268V^ is not completely refractory to Hdi or due to another function of Hdi unrelated to FtsZ. Taken together, our results indicated that Hdi has a physiological role in conferring a competitive advantage to the T5 phage through inhibition of FtsZ and consequently, cell division.

## DISCUSSION

In this work, we employed a genetic approach—high-throughput DNA sequencing of resistant bacterial mutants—to identify the target of Hdi. This phage T5 gene product inhibited bacterial growth when expressed from a plasmid, and during phage growth as well, Hdi targeted the cell division protein FtsZ, destabilizing ring formation and cell division, thus conferring a competitive advantage to the phage.

We previously showed that T7 phage small early protein Gp0.4 inhibits cell division by interacting with FtsZ (1). In the context of infection, inhibiting cell division during the early stage proves beneficial as it allows optimal utilization of bacterial resources. When cell division remains unrestricted, there is a possibility of a daughter cell escaping during the infection process, leading to a halved resource availability for the phage (Fig. 5A). Our research has demonstrated that for the T7 phage, this inhibition leads to an augmented number of progeny in each infection cycle, granting a competitive advantage (1). Consistent with previous findings, this phenomenon was also observed in the current study. On stationary-phase bacteria, the T7 phage lost its competitive advantage conferred by the division inhibitor, as expected due to lack of division (1). Unlike T7 phage, whose plaques continue to expand on stationary hosts (12), the T5 phage plaques do not continue to expand on stationary-phase bacteria. Therefore, an experiment using stationary-phase bacteria was not feasible in the current study. Nevertheless, we showed that on an FtsZ that is refractory to inhibition, the T5 phage, like the T7 phage (1), loses some of its competitiveness. We therefore conclude that the role of Hdi in the T5 phage is similar, i.e., it enables maximal use of host resources by preventing the escape of daughter cells (Fig. 5A).

Structure similarities were revealed using ColabFold (Mirdita, 2022 #5352) to predict the structure of Hdi in comparison to two other known FtsZ inhibitors, T7-0.4 protein (Kiro, 2013 #4690) and λ-Kil protein (Conter, 1996 #3714). All three short proteins encode two major alpha helices, with different lengths and angles between them (Fig. S5). This similarity suggests a similar mode of inhibitory action.

The Hdi inhibits FtsZ, but two positions in the FtsZ protein resist this inhibition: F268 and G191. Interestingly, a substitution that resists the natural FtsZ inhibitors MinCD and SulA was also identified at one of these positions (F268C) (13). Moreover, the F268V mutation also confers resistance to the T7 Gp0.4, which we discovered as inhibiting FtsZ (Fig. S6). The F268C mutant manifests lower GTPase activity; in all other respects, its properties are similar to the wild-type FtsZ (13). These findings suggest that position 268 harbors a key residue whose alteration can result in the evasion of different natural inhibitors while maintaining division capabilities.

From a therapeutic perspective, FtsZ is a target for developing new antimicrobial compounds because it is essential for cell division and is absent in eukaryotic cells. FtsZ possesses two distinct drug-binding regions, namely, the GTP binding site, located at the interface between its polymeric subunits, and the inter-domain cleft (IDC), nestled between the N-terminal and C-terminal segments of FtsZ’s core globular domain. The IDC contains the G191 residue identified in this study as crucial for T5.008 inhibition, also shown to be important for inhibition by several inhibitors (e.g., thiazole ring, naphthalene, and complex ring) (14). Notably, the IDC emerges as the favored binding site for the majority of anti-FtsZ molecules. In contrast, targeting the GTP binding site holds limited potential as an antimicrobial therapeutic due to its cytotoxicity on mammalian cells, stemming from the high sequence similarity shared with tubulin. Several studies have demonstrated the potential of FtsZ inhibitors as antibacterial agents. For example, Hu et al. (15) reported the synthesis and antibacterial activity of benzylamide derivatives that target FtsZ and inhibit the growth of *Staphylococcus aureus* and other Gram-positive bacteria.

Inhibition of FtsZ by a phage protein, as revealed in this study, strengthens our understanding of the molecular tools used by phages to manipulate and exploit host cells. The Hdi protein might be useful for studying bacterial cell division and as a new tool for combating antibiotic-resistant bacteria. Further studies need to be conducted to fully utilize Hdi as an antimicrobial compound: its minimal effective length for inhibition should be determined, and its FtsZ-inhibition capabilities across pathogenic bacterial species [it is noteworthy that Hdi was shown to inhibit three bacterial species other than *E. coli* (Fig. S1)], its stability inside and outside mammalian tissues, and its efficiency in penetrating bacteria should be explored. For these therapeutic applications, the mechanism of action of Hdi, and whether it directly interacts with FtsZ or whether its interactions are via other elements, should be addressed in future studies. We believe that the approach used here can be harnessed to reveal additional mechanisms used by phage proteins to inhibit bacterial cells, which may lead to the discovery of new targets for antibacterial drug design.

## MATERIALS AND METHODS

### Strains, media, and reagents

The *E. coli* strains used in this study are listed in Table S2. The strains were grown in Lysogeny Broth (LB; 1% wt/vol tryptone, 0.5% wt/vol yeast extract, and 0.5% wt/vol NaCl), 2xYT (1.6% tryptone, 1% yeast extract, and 0.5% NaCl), or Terrific broth (TB; 2.4% yeast extract, 1.2% tryptone, 0.94% wt/vol K_2_HPO_4_, and 0.02% wt/vol KH_2_PO_4_) at 37°C. Media were supplemented with 1.5% (wt/vol) agar to prepare solid plates. When necessary, media were supplemented with 35 µg/mL chloramphenicol, 50 µg/mL kanamycin, and 100 µg/mL ampicillin to maintain plasmids. To induce expression from inducible promoters, 0.1% or 0.2% (wt/vol) L-arabinose, 1 mM m-toluic acid, and 5 nM anhydrotetracycline (aTc) were added to the media. Restriction enzymes, ligation enzymes, DNA-modification enzymes, and Phusion High-Fidelity DNA Polymerase were from New England Biolabs.

### Plasmids

Plasmids were constructed using standard molecular biology techniques. DNA fragments were amplified by PCR. Standard DNA digestions and ligations were carried out according to the manufacturer’s instructions. Gibson Assembly (16) was carried out according to the New England Biolabs (NEB) protocol. Plasmids and primers that were used in this study are listed in Tables S3 and S4, respectively.

### Growth inhibition assay

To determine the effect of Hdi on bacterial growth (*E. coli NEB5α*, *Salmonella enterica*, *Shigella sonnei*, or *Enterobacter cloacae*), the bacteria harboring the indicated pBAD33-based plasmids were grown overnight in LB supplemented with chloramphenicol and 0.2% (wt/vol) D-glucose (to repress expression from the pBAD promoter) at 37°C. Overnight cultures were diluted 1:100 in fresh LB supplemented with chloramphenicol and grown at 37°C to an OD_600_ of 0.1. Cultures were then spotted on LB agar supplemented with chloramphenicol and either 0.2% D-glucose (repressive conditions) or 0.2% L-arabinose (inductive conditions). Note: a barely perceptible green line is present on a plate image in Figure 1, most likely due to the scanning process.

### Isolating resistant mutants

*E. coli* NEB5α harboring pBAD33-*hdi* was grown overnight in LB supplemented with chloramphenicol and 0.2% D-glucose at 37°C. The cultures were then pelleted and resuspended in the same volume of LB supplemented with chloramphenicol (3 mL), from which 100 µL was spread on a LB agar plate supplemented with chloramphenicol and 0.2% L-arabinose. Plates were incubated overnight at 37°C, and resistant colonies were collected.

Each independent resistant colony, isolated in an independent selection experiment to avoid sibling mutants, was transferred to 1.5 mL LB supplemented with chloramphenicol and 0.2% L-arabinose and grown at 37°C overnight. Plasmid DNA was extracted from these colonies as described previously (2). Naïve *E. coli* cells were transformed with 1 µL of the plasmid extractions and plated on LB agar plates supplemented with chloramphenicol and 0.2% D-glucose. The resultant colonies were picked and subjected to the growth inhibition assay detailed above.

### High-throughput DNA sequencing and analysis

Cultures from the seven mutants that were resistant to Hdi were combined, and their genomic DNA was extracted using the NucleoSpin Tissue Kit (Macherey-Nagel). The genomic DNA was processed using an Illumina Kit (Cat. No. 5025064) according to the manufacturer’s instructions. Sequencing was carried out using Illumina HiSeq 2500 in rapid mode, with single-read runs of 100 bp. The average DNA coverage per resistant strain was 95 reads per base pair. The sequencing results were used to identify *E. coli* genes that are putative targets of Hdi. The 100-bp reads were aligned to the reference sequence of the *E. coli* strain K-12 genome (NC000913) using Bowtie (version 2.0) (15). The alignment was visualized using SAMTools (14). Further bioinformatics analyses were carried out as described previously (2).

### Validation of high-throughput analysis by Sanger sequencing

Each independent mutant that was resistant to *hdi* expression was streaked on a LB agar plate supplemented with chloramphenicol and 0.2% L-arabinose. A single colony from this plate was picked, and its DNA served as the template for amplification of the *ftsZ* gene using primers MM209F and MM209R (Table S4). The PCR product was purified and Sanger sequenced using the same primer set to validate the mutation predicted by high-throughput sequencing.

### Construction of *E. coli ftsZ* mutant using the MAGE system

*E. coli* NEB5α and *E. coli* K-12 ftsZ-mNeon cells harboring pORTMAGE-Ec1 plasmid (17) were grown overnight in LB supplemented with 50 µg/mL kanamycin. These cultures were then diluted 1:100 in 25-mL fresh LB supplemented with kanamycin and grown at 37°C. Upon reaching an OD_600_ of 0.3–0.4, the cells were induced with 1 mM m-toluic acid for 30 min at 37°C. The bacteria were then harvested and washed twice with ice-cold water to make them electrocompetent. Electroporation was carried out with a 1-µL aliquot of 100 µM of the indicated 90-nt long MAGE phosphorothioate oligonucleotides (Table S4). After 1 h of recovery in prewarmed TB medium at 37°C, the bacteria were grown overnight in LB media supplemented with kanamycin at 37°C. They were then serially diluted and plated on LB plates. Approximately 10 colonies were screened for the desired mutation using Sanger sequencing with the appropriate primer pairs. The pORTMAGE plasmids were then cured from the bacteria. The colonies that were eventually used were validated by Sanger sequencing.

### Deletion of T5 *hdi* using lbuCas13a

*E. coli* BW25113 harboring pGEM-*hdi*^OH^ plasmid (Table S3) encoding homologous sequences for recombination flanking the *hdi* gene were grown overnight in LB supplemented with 100 µg/mL ampicillin. The culture was then diluted in LB supplemented with 1 mM MgSO_4_, 1 mM CaCl_2_, and 100 µg/mL ampicillin and aerated at 37°C. Upon reaching an OD_600_ of 0.4–0.5, the cells were infected with T5 at a multiplicity of infection of 0.1 and grown until lysis. The resulting lysates containing the wild-type and recombinant T5 phages were purified with chloroform and centrifuged. Counterselection for T5Δ*hdi* recombinant phage was carried out using the obtained lysates to infect an overnight culture of BW25113 cells harboring ptet-lbuCas13a plasmid and a plasmid encoding an efficient spacer against *hdi*. The cells were mixed with 0.7% molten agar supplemented with 5 nM aTc, 1 mM MgSO_4_, 1 mM CaCl_2_, 35 µg/mL chloroform, and 100 µg/mL ampicillin and poured on a 1.5% LB agar plate. The next day, several plaques were screened with PCR primer pairs amplifying the upstream and downstream sequences of *hdi* (Fig. S7). A phage identified by PCR as containing the deletion was further validated by Sanger sequencing.

### Time-lapse fluorescence microscopy

To visualize the effect of *hdi* on FtsZ rings using fluorescence microscopy, *E. coli* K-12-ftsZ mNeon and *E. coli* K-12-ftsZ^F268V^ mNeon plasmids containing arabinose-inducible pBAD33*-hdi* and pBAD33 were grown overnight at 37°C in LB supplemented with chloramphenicol and 0.2% D-glucose. Cultures were then diluted 1:100 in 3 mL of fresh LB supplemented with chloramphenicol and 0.2% D-glucose and incubated at 37°C until they reached an OD_600_ of 0.3. Cells were washed twice with fresh LB to remove the glucose and were resuspended in 3 mL LB supplemented with chloramphenicol. An aliquot of each cell suspension (2 µL) was spotted onto 1% (wt/vol) LB-agarose pads supplemented with 0.1% L-arabinose to induce expression from pBAD plasmids, which were placed face-down in 35 mm glass-bottom Cellview cell culture dishes. Time-lapse imaging was carried out with a Nikon Eclipse Ti2-E inverted motorized microscope equipped with a CFI PLAN apochromat DM 100X oil lambda PH-3 (NA, 1.45) objective lens, Lumencor SOLA SE II 395 light source, and DS-QI2 mono cooled digital microscope camera (16 MP). An ET-EGFP filter set (#49002) was used to visualize mNeon signals. Images were captured every 1 min for 60 min. The captured images were further processed using Fiji ImageJ suite (18).

To monitor the status of the FtsZ ring over time upon T5 phage infection using fluorescence microscopy, *E. coli* K-12-ftsZ mNeon and *E. coli* K-12-ftsZ (F268V) mNeon cells were grown overnight at 37°C in LB. Cultures were then diluted 1:100 in 3 mL of fresh LB and incubated at 37°C until they reached an OD_600_ of 0.3. The cells were infected with wild-type T5 or T5Δ*hdi* phage at a MOI of ~20. Immediately after mixing by pipetting, 2 µL of cell suspension was spotted on 1% LB-agarose pads and visualized under the microscope as described above. Images were captured every 30 s for 20 min.

### T5 wild-type and T5Δ*hdi* phage competition assay

*E. coli* K-12-ftsZ mNeon and *E. coli* K-12-ftsZ^F268V^ mNeon cells were grown overnight at 37°C in LB. Cultures were then diluted 1:100 in fresh LB supplemented with 1 mM MgSO_4_ and 1 mM CaCl_2_ and incubated at 37°C until they reached an OD_600_ of 0.4–0.5. The competition cycle was initiated by infecting the logarithmic culture with an equal mixture of wild-type T5 and T5Δ*hdi* at a MOI of 0.1. The infected cultures were grown at 37°C until lysis. The obtained lysates were diluted 1:1,000 in freshly growing logarithmic cultures for the next cycle. The relative abundance of wild-type T5 and T5Δ*hdi* in each cycle was determined by PCR amplification of the T5 genomic region flanking *hdi* using primers TM476F and TM476R (Table S4). The amplified products for wild-type T5 and T5Δ*hdi* were 394 bp and 271 bp, respectively. Band intensities were quantified using ImageJ software. The relative band intensity of each phage was calculated using the following formula: [(band corresponding to wild-type T5 or T5Δ*hdi*)/(sum of the band intensities)] × 100. The data were plotted using GraphPad Prism software. The statistical significance was calculated by unpaired *t*-test.

## SUPPLEMENTAL MATERIAL

Supplemental material spectrum.01697-23-s0001.docx Fig. S1 to S7, Tables S1 to S4, and legends for Movies S1 to S7.

Movie S1 spectrum.01697-23-s0002.avi Series of frames taken for the experiment of E. coli-ftsZ mNeon with pBAD33 shown in Fig. 3.

Movie S2 spectrum.01697-23-s0003.avi Series of frames taken for the experiment of E. coli-ftsZ mNeon with pBAD33-hdi shown in Fig. 3.

Movie S3 spectrum.01697-23-s0004.avi Series of frames taken for the experiment of E. coli-ftsZF268VmNeon with pBAD33 shown in Fig. 3.

Movie S4 spectrum.01697-23-s0005.avi Series of frames taken for the experiment of E. coli-ftsZF268VmNeon with pBAD33-hdi shown in Fig. 3.

Movie S5 spectrum.01697-23-s0006.avi Series of frames taken for the experiment of E. coli-ftsZ mNeon infected with WT-T5 shown in Fig. 4.

Movie S6 spectrum.01697-23-s0007.avi Series of frames taken for the experiment of E. coli-ftsZ mNeon infected with T5Δhdi shown in Fig. 4.

Movie S7 spectrum.01697-23-s0008.avi Series of frames taken for the experiment of E. coli-ftsZF268VmNeon infected with WT-T5 shown in Fig. 4.

ASM does not own the copyrights to Supplemental Material that may be linked to, or accessed through, an article. The authors have granted ASM a non-exclusive, world-wide license to publish the Supplemental Material files. Please contact the corresponding author directly for reuse.
