# Supplementary material for: Inhibition of host cell division by T5 protein 008 (Hdi)
Source: Microbiol Spectr. 2023 Oct 27;11(6):e01697-23. doi: 10.1128/spectrum.01697-23 (PMC10714956; doi:10.1128/spectrum.01697-23)
Supplement: Supplemental material — Fig. S1 to S7, Tables S1 to S4, and legends for Movies S1 to S7. [file spectrum.01697-23-s0001.docx]

**Supplementary Information for**

**Inhibition of Host Cell Division by T5 protein 008**

**Tridib Mahata^a^, Shahar Molshanski-Mor^a^, Moran G. Goren^a^, Miriam Kohen-Manor^a^, Ido Yosef^a^, Oren Avram^b^, Dor Salomon^a^, Udi Qimron^a,1^**

^a^*Department of Clinical Microbiology and Immunology, Sackler School of Medicine, Tel Aviv University, Tel Aviv 69978, Israel.*

^b^*The Shmunis School of Biomedicine and Cancer Research, George S. Wise Faculty of Life Sciences, Tel Aviv University, Tel Aviv 69978, Israel*

^1^Corresponding author; *Department of Clinical Microbiology and Immunology, Sackler School of Medicine, Tel Aviv University, Tel Aviv 69978, Israel*. Tel.: 972-3-6405191; Fax: 972-3-6409511; E-mail: ehudq@post.tau.ac.il

**Supplementary Figures**

**
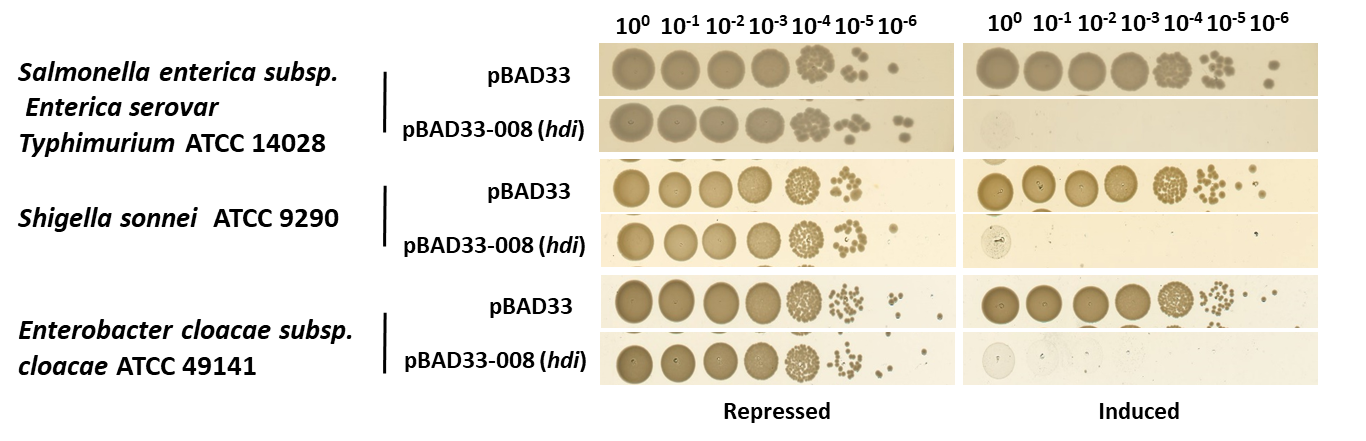
**

**Figure S1. T5.008 inhibition of different bacterial growth.** (A) The indicated bacterial species transformed with a plasmid encoding the T5.008 or with the control pBAD33 vector were inoculated on LB agar plates supplemented with 0.2% D-glucose (Repressed) or 0.2% L-arabinose (Induced). Results of one representative experiment out of three are shown.


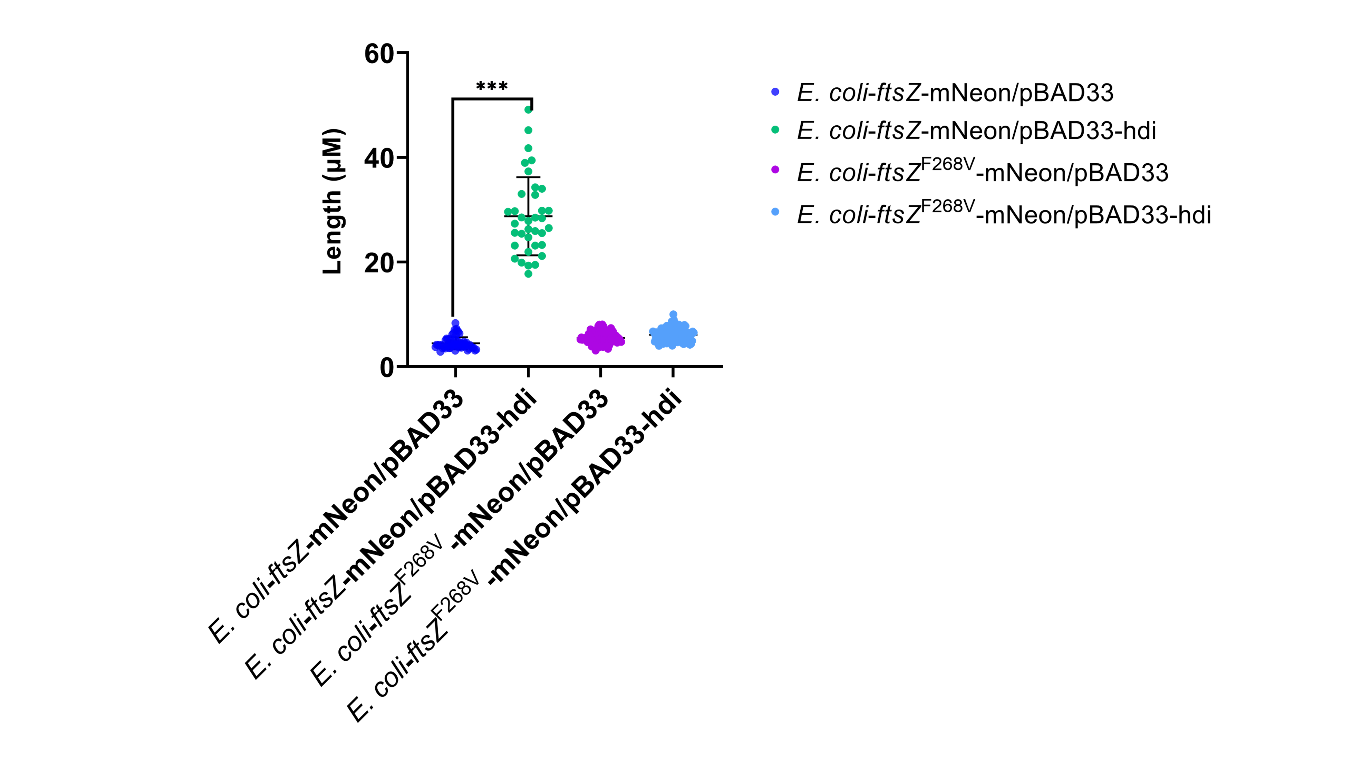


**Figure S2. Length measurement of the indicated bacterial cells with the inicated plasmids.** Lengths were measured using NIS-Elements AR Analysis software. Circels represent individual measurements, vertical line represents the mean ± SD [*E. coli*-*ftsZ*-mNeon/pBAD33 (n=61), *E. coli*-*ftsZ*-mNeon/pBAD33-hdi (n=34), *E. coli*-*ftsZ*^F268V^-mNeon/pBAD33 (n=91) and *E. coli*-*ftsZ*^F268V^-mNeon/pBAD33-hdi (n=101)]. *** *P* = 0.0001, as determined by unpaired, two-tailed Student’s *t* test.


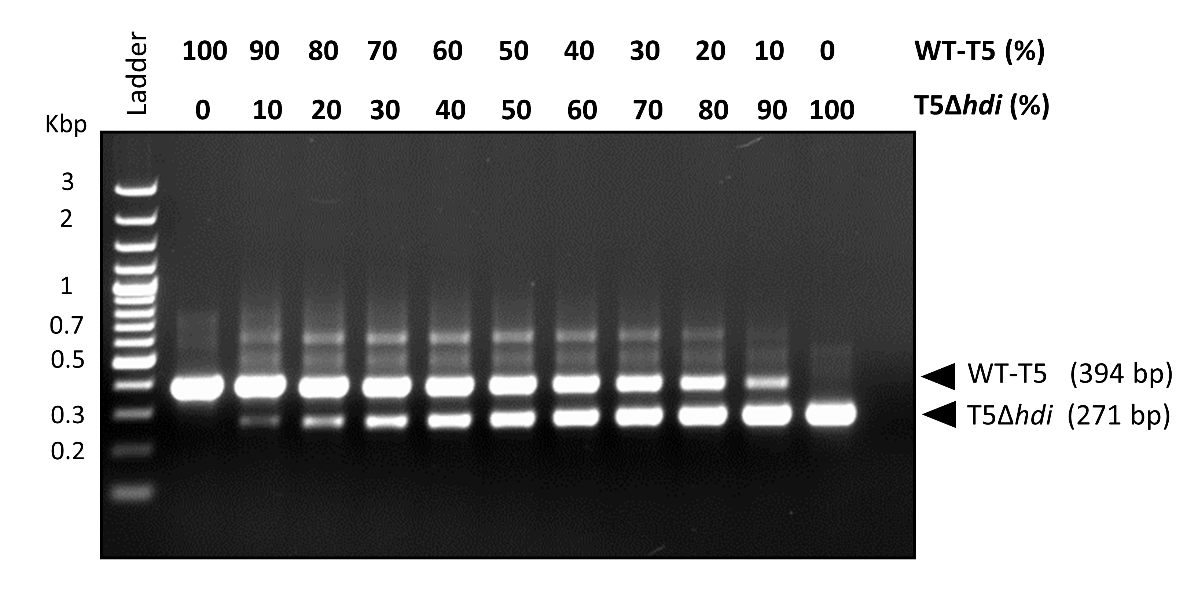


**Figure S3. Detecting phage levels by PCR.** PCR amplifying the region flanking the *hdi* gene was carried out on wt-T5 phage and on T5Δ*hdi* mixed at the indicated ratios. The upper bands are products obtained from amplifying the DNA of wt-T5 and the lower bands are from T5Δ*hdi*.


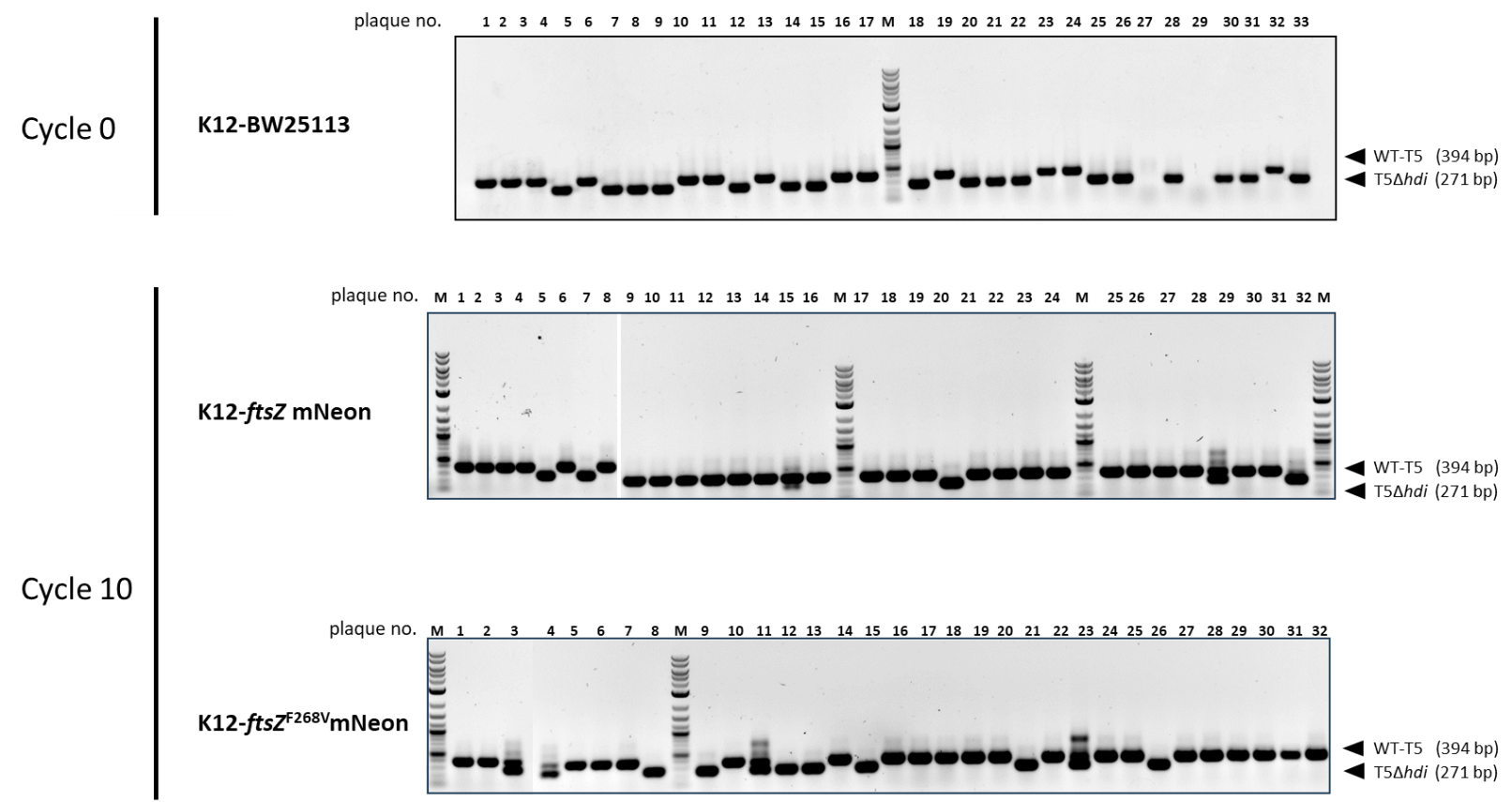


**Figure S4. Genotyping the plaques isolated from the indicated competition cycles.** Plaques for the competition initiation (cycle 0) were isolated from BW25113. Plaques were also taken after 10 competition cycles on K12-*ftsZ* mNeon and K12-*ftsZ*^F268V^mNeon. Individual plaques were genotyped for the wild type of deletion using primers TM476F and TM476R flanking the *hdi* gene.

**
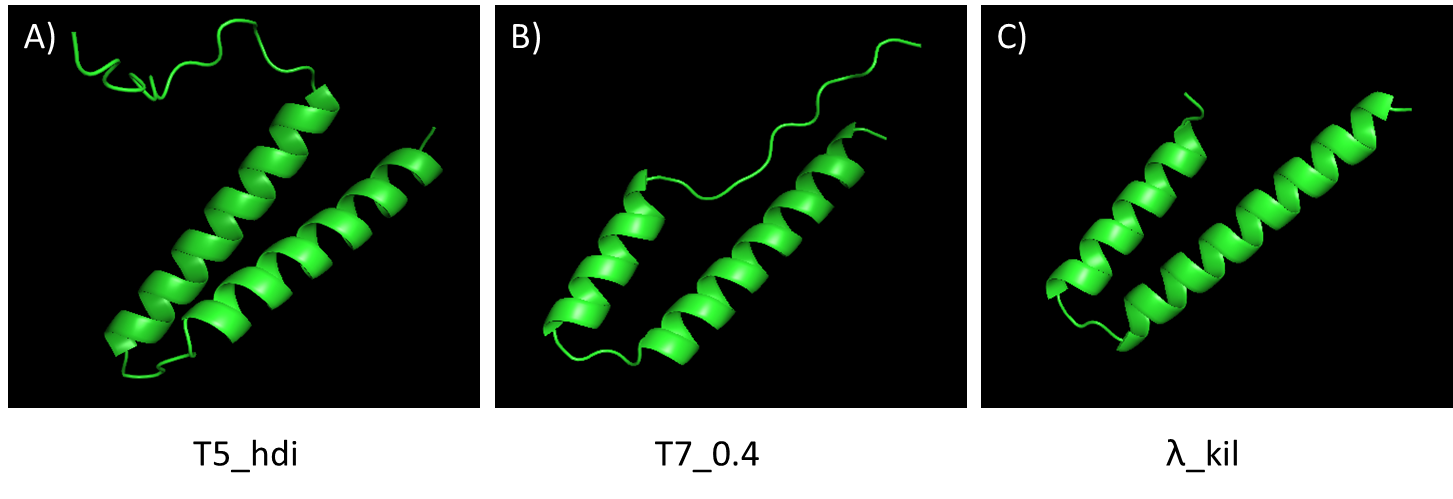
**

**Figure S5. Comparison of the structures of the FtsZ inhibitors T5_hdi (YP_006836.1) (A), T7_0.4 (NP_041955.1) (B), and λ_kil (WP_001700478.1) (C).** Structures were generated using ColabFold (1). The prediction was run with MMseqs2, 3 recycles, and with default seed. Models were plotted using pyMOL 2.5.0.

**
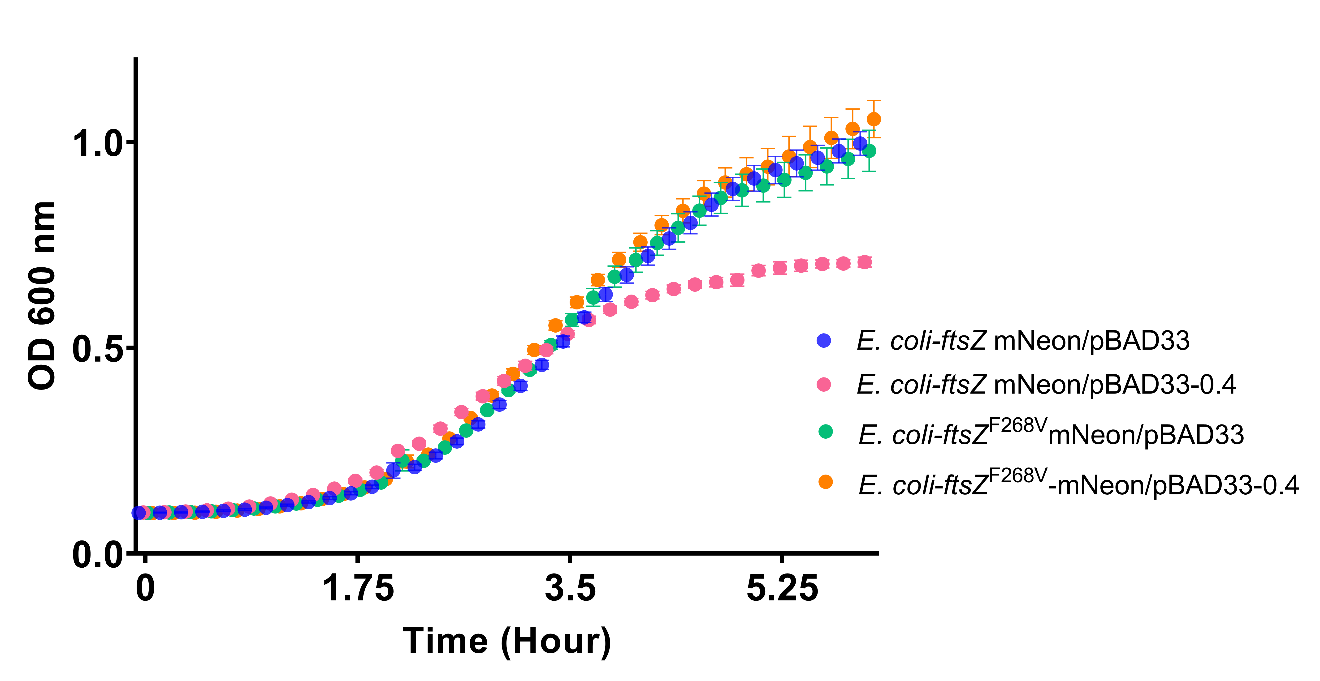
**

**Figure S6. Growth of bacteria in the presence of T7 Gp0.4.** Toxicity of T7 gene 0.4 as measured by growth of *E. coli* K-12-ftsZ mNeon or *E. coli* K-12-ftsZ^F268V^ mNeon containing an empty vector (pBAD33) or a plasmid for arabinose-inducible expression of T7-0.4 (pBAD33-0.4). Data represent the mean ± SD (n=3).

**
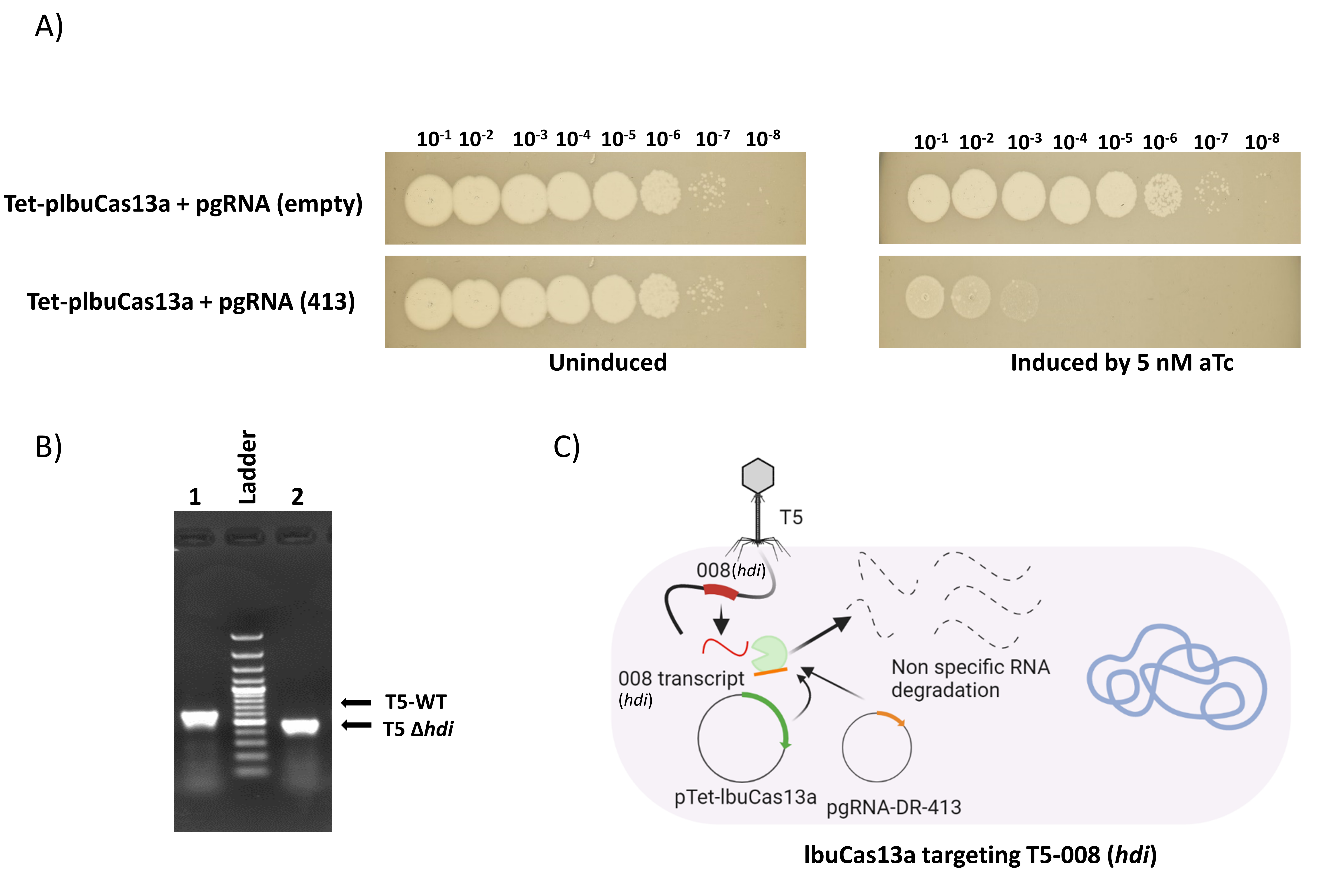
**

**Figure S7. Constructing T5 lacking *hdi*.** A) T5 phage dilutions were spotted on a lawn of BW25133 containing pTet-lbuCas13a and pgRNA-DR (empty vector) or pgRNA-DR-413. The cells were mixed with 0.7% molten agar supplemented with 5 nM aTc, 1 mM MgSO4, 1 mM CaCl2, 35 μg/mL chloroform and 100 μg/mL ampicillin, and poured on a 1.5% LB agar plate. B) The PCR amplified product of wt T5 (Lane 1) and T5 Δ*hdi* (Lane 2) using TM476F/R primer pairs separated on 1.5% agarose gel. C) Model of lbuCas13a mediated non-specific RNA degradation upon recognizing T5-008 (*hdi*) RNA transcript.

**Supplementary Tables**

**Table S1. Mutations detected in *ftsZ* of T5.008 (*hdi*)-resistant colonies**

| Resistant mutant | Type of mutation | Position of mutation within the genome^1^ | Position of Mutation within the *ftsZ* ORF | Nucleotide | Amino acid substitution^2^ |
| --- | --- | --- | --- | --- | --- |
| 1 | Substitution | 105875 | 191 | G->A | G191S |
| 2 | Substitution | 106107 | 268 | C->A | F268L |
| 3 | Substitution | 106105 | 268 | T->G | F268V |
| 4 | Substitution | 106105 | 268 | T->C | F268L |
| 5 | Substitution | 106105 | 268 | T->G | F268V |
| 6 | Substitution | 105875 | 191 | G->A | G191S |
| 7 | Substitution | 106106 | 268 | T->C | F268S |

^1^Numbering refers to *E. coli* strain K12 sub strain MG1655 genome, accession no. NC_000913.3
^2^Numbering refers to *E. coli* strain K12 sub strain MG1655, protein ID. NP_417075.1

**Table S2. *Bacterial* strains used in this study.**

| Strain | Genotype | Source |
| --- | --- | --- |
| *E. coli* Neb5α | *fhuA2Δ(argF-lacZ)U169 phoA glnV44 Φ80 Δ(lacZ)M15 gyrA96 recA1 relA1 endA1 thi-1 hsdr17* | NEB |
| *E. coli* BW25113 | *lac*I^q^, rrnBT14, Δ*lacZ*wJ16, *hsd*R514, Δ*ara*BAD AH33, Δ*rha*BAD LD78 | (2) |
| *E. coli* Neb5α *ftsZ*^F268V^ | *fhuA2Δ(argF-lacZ)U169 phoA glnV44 Φ80 Δ(lacZ)M15 gyrA96 recA1 relA1 endA1 thi-1 hsdr17,* ***ftsZ*^F268V^** | Used in this study |
| *E. coli* k12 *ftsZ*-mNeon | F- lambda- *ilvG*- *rfb*-50 *rph*-1, *ftsZ*-mNeon | (4) |
| *E. coli* k12 *ftsZ*^F268V^-mNeon | F- lambda- *ilvG*- *rfb*-50 *rph*-1, *ftsZ*^F268V^-mNeon | Used in this study |
| *E. coli* k12 *ftsZ* ^F268Y^-mNeon | F- lambda- *ilvG*- *rfb*-50 *rph*-1 *ftsZ*^F268Y^-mNeon | Used in this study |
| *Salmonella Enterica serovar Dublin TYT 3627* |  | Lab Collection |
| *Shigella sonnei  ATCC 9290* |  | ATCC |
| *Enterobacter cloacae subsp.*  *cloacae ATCC 49141* |  | ATCC |

**Table S3. List of plasmids used in this study.**

| Plasmids | Comments |
| --- | --- |
| pBAD33 | L-arabinose inducible expression vector, p15A origin, chloramphenicol resistance |
| pBAD33-008 (*hdi*) | T5 gene 008 (*hdi*) cloned between SalI and PstI sites of pBAD33 MCS |
| pBAD33-0.4 | T7 gene 0.4 cloned between SalI and PstI sites of pBAD33 MCS |
| pGEM-T easy vector | Cloning vector |
| pGEM-*hdi*^OH^ | pGEM-T easy vector with *hdi* overhang was built by blunt end ligation. |
| pORTMAGE-EC1 | Encode highly efficient recombinase cspRecT and used for constructing ftsZ mutants. |
| pSUMO-lbucas13a | SUMO bacterial expression vector. (Addgene #115267) |
| pTet-lbucas13a | Tet inducible lbuCas13a expression vector. |
| pgRNA-DR | Plasmid with direct repeat for spacers. |
| pgRNA-DR-413 | Plasmid encode *hdi* targeting spacer. |

**Table S4. List of primers used in this study.**

| Primer | Sequence (5’-3’) | | Comments |
| --- | --- | --- | --- |
| SM44F5 | ATATGTCGACTATAAGGAATCGACGATATG | | Amplify T5 gene 008 (*hdi*) and sub-clone it between SalI and PstI sites of pBAD33 to construct pBAD33-008 (*hdi*) |
| SM44R5 | ATTACTGCAGTGTCAAAC TATTTTTTGC AG | |  |
| SM24F15 | ATATGTCGACGAGGAGGATGAAGAGTAATG | | Amplify T7 gene 0.4 and sub-clone it between SalI and PstI sites of pBAD33 to construct pBAD33 -0.4 |
| SM24R15 | ATTACTGCAGTCACTCAGCAGATTCTAAAG | |  |
| MM209F | ATTATTCGACGGCGGTGGGA | | Validate *ftsZ* mutations using Sanger sequencing |
| MM209R | CAGGGTGACTTTCTTGCCGGT | |  |
| TM402F | GATGTAGAGGAAGTTCTATAACACCCTAGC | | Amplify the pGEM-T-009-BC Pone-007 to construct pGEM-*hdi*^OH^ |
| TM33R | GTACCGAGCTCGAATTCGCTAGC | |  |
| TM464R | AGATCCTTTCTCCTCTTTAGATCTTTTGAATTC | | Amplify pdCas9 to construct ptet-lbuCas13a using Gibson assembly. |
| IY726 | TTAGAAAAACTCATCGAGCATCAAATG | |  |
| TM465R | CCTCAGGGCCGGATCCGTAT | | Amplify lbuCas13a from pSumo-lbucas13a backbone to construct ptet-lbuCas13a using Gibson assembly. |
| TM466F | AGGCCTATGCGGCCGCTAAG | |  |
| TM475F | ATCCCCGGGTTAATTAAGATGAAGCGACGATG | |  |
| TM475R | CATCCTCCTCGAGCTCGAATTCGCTAG | |  |
| TM413F | GTGGCAGTACAAGAAAGGCCGCCAGCAAGGTC | | To construct pgRNA-DR-413 using golden gate assembly. |
| TM413R | AAACGACCTTGCTGGCGGCCTTTCTTGTACTG | |  |
| TM476F | GGACACATTATGGTAAGTGCCGAC | | To verify T5-*hdi* deletion using Sanger sequencing. |
| TM476R | GAACTACACGGCGGGAAGTTTAC | |  |
| TM281R | | C*G*CGTTGTCGGAAGCAAATGCACGGATGGTGTTACCTACCGTTTCGAACTCATCCAGACGCAGGTCGACGCCCGCCGTGATGTTAACC*A*G | Used for pORTMAGE mediated mutation of ftsZ F268V |
| TM363R | | C*G*CGTTGTCGGAAGCAAATGCACGGATGGTGTTACCTACCGTTTCGAACTCATCCAGACGCAGGTCGTAGCCCGCCGTGATGTTAACC*A*G | Used for pORTMAGE mediated mutation of ftsZ F268Y |

***** Phosphothioate linkage

**Supplementary References**

1. Mirdita M, Schütze K, Moriwaki Y, Heo L, Ovchinnikov S, Steinegger M (2022) ColabFold: making protein folding accessible to all. *Nature Methods* 19(6):679-682.

2. Baba T, Ara T, Hasegawa M, Takai Y, Okumura Y, Baba M, Datsenko KA, Tomita M, Wanner BL, Mori H (2006) Construction of Escherichia coli K-12 in-frame, single-gene knockout mutants: the Keio collection. *Mol Syst Biol* 2:1-11.
